# Self-Schema, Attachment Style, and Treatment Outcome of Patients in an Opiate Maintenance Treatment Unit

**DOI:** 10.3389/fpsyg.2021.595883

**Published:** 2021-11-23

**Authors:** Emelie Hovelius, Ellen Lindén, Hans Bengtsson, Anders Håkansson

**Affiliations:** ^1^Malmö Addiction Centre, Region Skåne, Malmö, Sweden; ^2^Department of Psychology, Lund University, Lund, Sweden; ^3^Department of Clinical Science, Lund University, Lund, Sweden

**Keywords:** opiate maintenance treatment, methadone, buprenorphine, attachment style, self-schema, YSQ-S, YPI, ECR-RS

## Abstract

The aim of this study was to explore self-schemas and attachment style among patients in a methadone or buprenorphine maintenance treatment program of opiate dependence, in relation to treatment outcome (relapse in substance use). The study included 84 patients (21 women and 63 men) in a psychiatric clinic in Malmö, Sweden, providing maintenance treatment of opiate dependence. Three self-report instruments were employed, Young Schema Questionnaire Short version (YSQ-S) and Young Parenting Inventory (YPI) for studying self-schemas and Experiences in Close Relationships–Relationship Structures questionnaire (ECR-RS) for studying attachment style. Demographical data and relapse in substance abuse were registered. The study demonstrated, unsurprisingly, that an insecure attachment style was more common in the group of patients compared to available general population reference data. Significant correlations were found between attachment style and core beliefs about the self (self-schemas). Memories of parenting experiences from childhood (YPI) showed correlations with ongoing self-schemas (YSQ-S). Treatment outcome, defined as relapses in substance abuse, was associated to a minor degree with self-schemas but showed no correlation with attachment style. Patients who did not work or study had more maladaptive self-schemas and insecure attachment style, and a higher incidence of relapse in abuse than patients who were working or studying.

## Introduction

The abuse of opiates creates problems at both individual and community levels. It is therefore important to understand how psychological factors contribute to such abuse and affect the possibility of reducing this through treatment. Previous research on opiate abuse has suggested a number of such factors (Darke et al., 2017; Strang et al., 2020). The present study focuses on cognitive and emotional structures that affect the view of the self and others. More specifically, we map self-schemas and attachment style in a group of opiate dependent patients in an opiate maintenance treatment unit and examine the relation between these structures and relapse in abuse.

The study of self-schema is based on cognitive theory and describes specific assumptions about oneself and one’s personal characteristics and skills. These assumptions are fundamental and often deeply established in an individual, despite often not being well expressed to the individual herself. Cognitive theory conceptualizes schemas as unaware cognitive structures that influence the processing of information (perception, coding, and deriving information) and how events are interpreted (Beck, 2011). Individuals typically perceive their self-schema as representing the truth and tend to endorse information that confirms these early schemas, whereas information in conflict with existing schemas is neglected or rejected (Welburn et al., 2002). For these reasons, early and maladaptive schemas tend to be self-confirming and resistant to change (Schmidt et al., 1995; Brotchie et al., 2004).

An important assumption in the cognitive theory about self-schemas is that vulnerable individuals develop negative, maladaptive, and depressive self-schemas as a response to unfavorable experiences lived early in life. Even after being inactive during a period in life, latent negative self-schemas may be activated by negative life events and lead to the development or maintenance of psychological distress, such as depressive symptoms (Franck et al., 2008; Dozois et al., 2009; Seeds and Dozois, 2010). Self-schemas may also be differentially activated by mood (Stopa and Waters, 2005).

Several studies suggest that drug dependence is associated with negative self-schemas. Tarquinio et al. (2001), for example, noted a higher frequency of maladaptive self-schemas in individuals with alcohol dependence, compared to a control group. Avants et al. (1996) examined patients with cocaine dependence in methadone-maintained patients and found a higher degree of maladaptive self-schemas in ongoing substance use, compared to patients in abstinence. Of particular relevance for the current study, Brotchie et al. (2004) examined self-schemas in patients with alcohol, opioid, or combined alcohol and opioid abuse and compared them to a non-clinical control group, using the short version of the Young Schema Questionnaire (YSQ-S). All clinical groups were found to have a higher frequency of maladaptive self-schemas, compared to the non-clinical group. The content of the negative self-schemas also differed across the three clinical groups. Overall, difficulties were somewhat more pronounced in patients with alcohol abuse than in patients with opioid abuse. Based on their findings, the authors speculate that alcohol abuse may differ from opioid abuse with respect to the style of information processing; alcohol drinking may reduce the experience of negative affect once it has been activated by a maladaptive self-schema, whereas opioids may reduce activation of the maladaptive self-schemas.

Aspects of self-schemas may be integrated into internal working models of attachment (Seeds and Dozois, 2010). Bowlby (1969, 1988) described internal working models of attachment as cognitive and emotional structures evolving from early relational experiences with caregivers. These models are believed to be relatively stable across time and affect how new relational experiences are processed, interpreted, and understood (Gross et al., 2017; Reisz et al., 2018). Thus, they continue to play a crucial role in an individual’s perceptions and attitudes to close relationships (Bowlby, 1988; Ainsworth, 1991).

Attachment models include basic assumptions about oneself, others, and one’s relations to others. Bartholomew and Horowitz (1991) developed a model of four attachment styles derived from different combinations of positive or negative experience of oneself and others. These styles are referred to as secure (positive self and positive other), preoccupied (negative self and positive other), dismissing (positive self and negative other), and fearful (negative self and negative other; Cassidy and Shaver, 1999; Gross et al., 2017; Reisz et al., 2018). Shorey and Snyder (2006) have studied the association between attachment and psychopathology in adults. In several studies, patients with drug dependence are described to have a higher degree of insecure attachment than individuals without drug dependence (e.g., Andersson and Eisemann, 2004; Schindler et al., 2005; Caspers et al., 2006; Potik et al., 2014; Schindler, 2019). However, to our knowledge, there is a need of more specifically addressed studies of self-schema and attachment style of patients in an opiate maintenance treatment unit. In addition, given the severe clinical course seen in active opioid use disorders, there is a reason to study how self-schema and attachment style may be associated to treatment outcome, described as relapse into illicit substance use.

Researchers traditionally have assumed that internal working models affect a broad range of different relational domains and that an individual’s attachment style remains the same across different types of relationships (Hazan and Shaver, 1987; Bowlby, 1988; Bartholomew and Horowitz, 1991). However, Baldwin et al. (1996) demonstrated that within an individual, there is likely a variability in expectations and assumptions in relation to significant others. Therefore, in the current study, attachment style was measured using the Experiences in Close Relationships-Relationship Structures questionnaire (ECR-RS; Fraley et al., 2011a), which provides information about variability in individuals’ attachment style across different significant relationships. Thus, by using the ECR-RS, it is possible to assess whether an adult individual presents different attachment styles specific to separate personal relationships. Attachment styles may be conceptualized in a two-dimensional way: lower anxiety in combination with lower avoidance represents a *secure* attachment style, lower avoidance and higher anxiety represents a *preoccupied*, lower anxiety and higher avoidance represents a *dismissing-avoidant*, and higher on both anxiety and avoidance represents a *fearful-avoidant* attachment style. People with a preoccupied attachment style tend to fear rejection and worry about whether others love them. People with dismissing-avoidant attachment style are less comfortable in opening up to others and depending on others. People with fearful-avoidant attachment style are uncomfortable in depending on others and are also worried that others may not be emotionally available when they are most needed (Fraley et al., 2006, 2011a).

### Aims of the Study

The current study examined self-schemas and attachment style in patients with ongoing opioid maintenance treatment for an opioid use disorder. The investigation had three main aims. One aim was to provide more information about how the self-schemas and attachment style of individuals with opiate dependence differ from available reference data from non-clinical samples. Based on prior research (Brotchie et al., 2004), we anticipated that opiate dependence would be associated with negative self-schemas at least in some areas. The results of the current study may contribute to specifying the nature of those areas. Further, based on prior drug-related attachment research, we anticipated that opiate dependence in the current sample would be associated with an elevated level of insecure attachment style compared to what has been reported in non-clinical populations. By using an assessment tool that recognizes the possibility of variability in attachment security across relationships, the current investigation examines whether this insecurity is limited to certain relationships or is of a more general nature. A second study aim was to examine whether individual differences in the assessed variables were consistent and conformed to theoretical predictions. It was hypothesized that the patients’ description of childhood caregiving experiences would be congruent with their current attachment style and self-schemas. The third main aim of the study was to examine whether individual differences in self-schemas or attachment styles would correlate with treatment outcome (relapse in substance use) and with sociodemographic data. We hypothesized that insecure attachment style and/or maladaptive self-schemas would be positively and significantly associated with relapse in opiate use during the treatment.

## Materials and Methods

### Participants

Male (*n*=63) and female (*n*=21) patients above 20years of age with opioid dependence and an ongoing opiate maintenance treatment (OMT, Socialstyrelsen, 2015) with methadone or buprenorphine at an OMT unit in Malmö, Sweden, participated in the study. Patients were recruited by personnel posters and folders at the unit. Interested patients were provided with written information about the study and were left with time to consider their possible participation. The mean age was 44.7years (median 43.5years, range 25–65years). Of the participants, 32 were in work (employment or study) and 52 were in sick-leave or social welfare; 35 had a partner and 49 were single; and 64 were Swedes and 20 had other cultural background. Participants had been included in OMT at the unit during at least 4months. Among 133 patients eligible for inclusion, 24 patients refused participation (20 men and four women, aged 34–63years, mean age 49.9years). Patients were excluded from the study in case of severe language difficulties, severe cognitive difficulties, or severe mental health or physical disorders (*n*=25, 24 men, and one woman, aged 29–65years, mean age 48.5years). The evaluation of exclusion criteria was carried out by a psychologist (the first author of the present paper) in collaboration with the team of the OMT facility. Participants received a gift card with a value corresponding to approximately nine euros.

### Instruments

Three self-report questionnaires were administered: the 75-item Young Schema Questionnaire: Short version (YSQ-S; Young, 1998), the 72-item Young Parenting Inventory (YPI, Young, 1999), and the 36-item Experiences in Close Relationships-Relationship Structures questionnaire (ECR-RS, Fraley et al., 2011a; Moreira et al., 2015). In addition to completing the three self-report questionnaires, participants were asked to report age, gender, marital status, sources of income, time in OMT, and nationality/cultural background.

*The Young Schema Questionnaire Short version* (YSQ-S) is based on the Young’s cognitive theory about potential maladaptive schemas developed early in life in vulnerable individuals and is a briefer version of the Young Schema Questionnaire (Schmidt et al., 1995). The YSQ-S assesses the presence of 15 different maladaptive schemas: Emotional deprivation, Abandonment, Mistrust/abuse, Social isolation, Defectiveness/shame, Failure to achieve, Functional dependence/incompetence, Vulnerability to harm and illness, Enmeshment, Subjugation, Self-sacrifice, Emotional inhibition, Unrelenting standards, Entitlement, and Insufficient self-control/−discipline. To illustrate, a sample item for Abandonment is: “I worry that people I feel close to will leave me or abandon me,” and for Mistrust/abuse: “I feel that I cannot let my guard down in the presence of other people, or else they will intentionally hurt me.” Statements are responded to along a 6-degree Likert scale, with higher values indicating more maladaptive self-schemes. The YSQ-S has shown to provide good psychometric data (Waller et al., 2001; Welburn et al., 2002; Baranoff et al., 2006). The established Swedish translation of the scale is available online (Carlbring et al., 1999). In the present study, reliability analyses of subscales in YSQ-S generated Cronbach’s alphas between 0.63 and 0.90.

*The Young Parenting Inventory* (YPI) is based on clinical experience and examines an individual’s memories of relational experience of close caregivers, in order to identify a total of 17 potential maladaptive assumptions: Emotional deprivation, Abandonment, Mistrust/abuse, Defectiveness/shame, Failure to achieve, Functional dependence/incompetence, Vulnerability to harm and illness, Enmeshment, Subjugation, Self- sacrifice, Emotional inhibition, Unrelenting standards, Entitlement, Negativity/pessimism, Punitiveness, Approval-seeking/recognition seeking, and Insufficient self-control/−discipline. To illustrate, an item for Abandonment is: “Withdrew or left me alone for extended periods,” and an item for Mistrust/abuse is: “Abused me physically, emotionally, or sexually.” The established Swedish version of the YPI is available online (Carlbring and Söderberg, 1999). In YPI, the subscales of Functional dependence/incompetence (Di), Self-sacrifice (Ss), and Entitlement (Et) resulted in low Cronbach’s alpha (*r*<0.60) and were therefore excluded in the study. Remaining subscales in the present study had Cronbach’s alpha between 0.62 and 0.95. The Cronbach’s alpha for separate sub-scales is reported to range from 0.67 to 0.92 (Sheffield et al., 2005). Self-schemas examined in the YPI and in the YSQ-S are overlapping to a large extent. However, the Social isolation schema is present only in the YSQ-S, and Negativity/pessimism, Punitiveness and Approval-seeking/recognition seeking only in the YPI (Sheffield et al., 2005, 2006). YPI statements are responded to along a 6-degree Likert scale, in two separate versions corresponding to maternal and paternal parenting style, respectively. Higher values reported indicate more maladaptive self-schemas.

*The Experiences in Close Relationships-Relationship Structures questionnaire* (ECR-RS) aims to determine attachment style in different relations based on nine items and has shown good psychometric characteristics (Fraley et al., 2011b; Alessandri et al., 2014; Busonera et al., 2014; Moreira et al., 2015; da Rocha et al., 2017). The ECR-RS provides a result on two factors: Anxiety (including abandonment and insufficient love) and Avoidance (avoidance of intimacy and emotional expressions). Fraley et al. (2006) argues that the attachment style of adults may be better described using the terminology of differences of degree in Anxiety and Avoidance. Higher rating on both scales represented a fearful-avoidant attachment style. In the present study, we follow this procedure and not the four categories (secure, preoccupied, dismissing-avoidant, and fearful-avoidant). Example of items included in studying the different relationships: “I do not feel comfortable opening up to this person.” The instrument has provided Cronbach’s alpha values ranging from 0.72 to 0.91 (Moreira et al., 2015). The Swedish ECR-RS version was developed for the current study from a translation of the English original version. Back-translation by a second individual who did not have access to the original version resulted in a satisfactory agreement with the original version. For ECR-RS, the reliability analysis in the present sample generated a Cronbach’s alpha between 0.74 and 0.92 for all independent scales. In the present study, patients’ attachment was examined for close relationships and, in general, for relations to mother/mother-like figure, father/father-like figure, and romantic partner. In case the patient did not have a partner, instructions were to respond with respect to a previous partner. ECR-RS items were answered using a 7-grade Likert scale.

### Study Procedures

In case of providing written informed consent to participate, the patients filled in the questionnaires and had the possibility to ask the first author for help in case of questions about the questionnaires. Participants were encouraged to take breaks and to ask for help whenever needed.

Treatment outcome was measured using the urine sample data for substance use during OMT. The clinical course of patients was observed and rated for 1year, divided into three 4-month periods: 4–8months prior to when they filled in the questionnaires, 0–4months prior to this point in time, and 4months after filling out the questionnaires. The clinical course was rated with respect to three aspects of treatment outcome: relapse in drug abuse with positive urine screens (scored 2), periods of abstinence and relapse (scored 1), and abstinence (scored 0). Scores were summed and averaged for the 1-year period for each patient (0–2).

The patient’s participation in the study was noted in the hospital records, but without any data describing the actual study data collected from the patient. Information about the course of treatment was collected from the first author and from a nurse assistant at the unit, with independent reporting from the two. In a few cases, where this reporting differed, the clinical course of treatment was discussed and resolved with the contact staff of the patient. The study was approved by the Regional Ethics Board in Lund university, Sweden (file number 2014/939).

### Data Analysis

Comparison group data were retrieved from three studies. For the ECR-RS, data were used from Moreira et al. (2015), an e-mail-recruited population study in Portugal (*n*=236; 169 females and 67 males, aged 18–66years) in which attachment style was studied in relation to mothers, fathers and partners, as well as best friends, yielding estimates of avoidance and anxiety for the separate independent scales. For YSQ-S, comparison group data was obtained from Stopa and Waters (2005), who conducted their study on a non-clinical group of students and employees at a British university (*n*=30; 13 men, mean age 24.4years, SD 4.3, and 17 women, mean age 24.1years, SD 10.8). The sample completed the YSQ-S on three different occasions: in neutral mood, and following happy and depressed mood inductions. Here, we used the group results derived from non-experimental (neutral) conditions. For the YPI, finally, we used as comparison group results presented by Atalay et al. (2008), based on a sample of hospital employees (*n*=45; 30 women, 15 men, aged 18–65years). Correlations between sub-scales, as well as correlations between separate items and the treatment outcome (relapse) variable, were calculated as binary correlations. T-tests were made for comparison between the study sample and the normative samples, with respect to each survey item. As significance level, we used *p*≤0.05 (two-sided) throughout the study.

## Results

As shown in Table 1, significant differences in attachment style were found between the patients and the comparison group on all scales, patients scoring higher (more insecure) than the comparison group. Significant differences in self-schemas (Table 2) as assessed with YSQ-S were obtained between patients and controls with respect to Abandonment, Mistrust/abuse, Social isolation, and Enmeshment, where patients scored significantly higher than controls. Finally, in YPI, both for maternal and paternal relationships, patients scored higher than controls on Abandonment, Mistrust/abuse and Failure to achieve, Defectiveness/Shame, and Punitiveness. In contrast, patients scored lower than controls on Vulnerability to harm and illness, Enmeshment, Negativity/pessimism, Emotional inhibition (for mother), and Approval-seeking/recognition-seeking (Table 2). Thus, in many, but not all, respects, self-schema tended to be more negative in the patient group.

**Table 1:** ECR-RS scales (mean scores and standard deviation) for patients in treatment for opiate addiction compared to normative values (non-clinical groups) and scales correlations with relapse in substance use during treatment (*r_p_*).

Attachment style	Patients group M (SD)	Norm group M (SD)	*t*	Relapse in substance use (*r_p_*)
Avoidance: General (AvG)	3.92 (1.15)	2.32 (0.75)	11.88^***^	0.18
Avoidance: Mother (AvM)	3.48 (1.53)	2.48 (1.24)	5.37^***^	−0.03
Avoidance: Father (AvF)	4.06 (1.60)	3.12 (1.56)	4.55^***^	−0.03
Avoidance: Partner (AvP)	3.05 (1.37)	1.62 (0.66)	9.09^***^	0.13
Anxiety: General (AnG)	3.16 (1.73)	2.12 (1.08)	4.86^***^	−0.09
Anxiety: Mother (AnM)	2.45 (1.64)	1.82 (1.21)	3.21^**^	−0.01
Anxiety: Father (AnF)	2.65 (1.53)	1.82 (1.28)	4.32^***^	0.12
Anxiety: Partner (AnP)	3.15 (1.68)	2.69 (1.53)	2.18^*^	0
Fearful-Avoidant: General (FAG)	3.54 (1.06)	n.a.	n.a.	0.03
Fearful-Avoidant: Mother (FAM)	2.97 (1.28)	n.a.	n.a.	−0.02
Fearful-Avoidant: Father (FAF)	3.35 (1.31)	n.a.	n.a.	0.05
Fearful-Avoidant: Partner (FAP)	3.10 (1.27)	n.a.	n.a.	0.07

*** *p≤0.001*;

** *p≤0.01*;

* *p≤0.05*.

*n.a., not available. ECR-RS scores for patients are based on n=78–84. ECR-RS-scores for norm group are retrieved from Moreira et al. (2015)*.

**Table 2:** YSQ-S- and YPI (M=mother/F=father) subscales (mean scores, standard deviation) for patients in treatment for opiate addiction compared to normative values (non-clinical groups) and subscales correlations with relapse in substance use during treatment (*r_p_*).

Self-schema	Patient group M (SD)	Norm group M (SD)	*t*	Relapse in substance use (*r_p_*)
YSQ				
Ed Emotional deprivation	2.35 (1.11)	2.11 (1.32)	0.89	0.08
Ab Abandonment	2.30 (1.22)	1.82 (0.82)	2.39^*^	0.12
Ma Mistrust/abuse	2.70 (1.28)	1.82 (0.73)	4.66^***^	0.18^†^
Si Social isolation	2.61 (1.29)	1.77 (0.99)	3.66^***^	0.20^†^
Ds Defectiveness /shame	1.74 (0.81)	1.47 (0.65)	1.82	0.06
Fa Failure to achieve	1.77 (1.00)	1.93 (1.22)	−0.65	0.08
Di Functional dependence/incompetence	1.72 (0.90)	1.63 (0.74)	0.54	0.11
Vh Vulnerability to harm & illness	2.25 (1.22)	1.72 (0.85)	0.62	0.18
Em Enmeshment	1.79 (0.94)	1.43 (0.77)	2.07^*^	0.24^*^
Sb Subjugation	2.02 (1.07)	1.92 (0.89)	0.5	0.17
Ss Self sacrifice	2.98 (1.18)	2.66 (0.98)	1.45	0.21^†^
Ei Emotional inhibition	2.10 (0.91)	1.95 (1.01)	0.72	0.27^*^
Us Unrelenting standards	2.95 (1.35)	3.35 (1.34)	−1.4	0.06
Et Entitlement	2.17 (1.05)	2.11 (0.84)	0.31	0.24^*^
Is Insufficient self-control/−discipline	2.59 (0.98)	2.76 (1.25)	−0.67	0.21^†^
YPI				
M-Ed Emotional deprivation	3.22 (1.59)	3.72 (1.58)	−1.71	−0.09
F-Ed Emotional deprivation	3.68 (1.39)	3.66 (0.95)	0.09	−0.11
M-Ab Abandonment	1.76 (1.11)	1.27 (0.64)	3.17^**^	−0.07
F-Ab Abandonment	2.38 (1.39)	1.18 (0.41)	7.07^***^	−0.03
M-Ma Mistrust/abuse	1.70 (1.20)	1.31 (0.63)	2.39^*^	0.01
F-Ma Mistrust/abuse	1.82 (0.98)	1.19 (0.40)	4.95^***^	0.04
M-Vh Vulnerability to harm & illness	2.17 (1.21)	2.64 (0.92)	−2.45^*^	0.30^**^
F-Vh Vulnerability to harm & illness	1.96 (1.16)	3.96 (0.87)	−10.69^***^	0.28^*^
M-Ds Defectiveness /shame	1.99 (1.29)	1.75 (0.83)	1.27	−0.07
F-Ds Defectiveness /shame	2.20 (1.37)	1.52 (0.59)	3.74^***^	−0.15
M-Fa Failure to achieve	1.81 (0.99)	1.42 (0.63)	2.64^**^	−0.02
F-Fa Failure to achieve	1.86 (1.01)	1.39 (0.54)	3.30^**^	0.08
M-Sb Subjugation	2.03 (1.30)	2.49 (1.27)	−1.94	0.01
F-Sb Subjugation	2.15 (1.27)	2.22 (1.15)	−0.31	−0.05
M-Us Unrelenting standards	2.53 (1.12)	2.53 (0.93)	0	0.07
F-Us Unrelenting standards	2.64 (1.14)	3.00 (1.01)	−1.79	0.07
M-Is Insufficient self-control/−discipline	2.14 (0.96)	n.a.	n.a.	0.17
F-Is Insufficient self-control/−discipline	2.24 (1.01)	n.a.	n.a.	0.28^*^
M-Em Enmeshment	1.72 (0.85)	3.68 (0.93)	−11.71^***^	0.11
F-Em Enmeshment	1.71 (0.87)	3.22 (0.99)	−8.48^***^	0.15
M-Np Negativity/pessimism	2.42 (1.26)	3.19 (1.01)	−3.80^***^	0.14
F-Np Negativity/pessimism	2.17 (1.14)	2.55 (0.92)	−1.99^*^	0.22^†^
M-Ei Emotional inhibition	2.78 (1.10)	3.15 (0.89)	−2.06^*^	0.09
F-Ei Emotional inhibition	2.98 (1.09)	3.07 (0.88)	−0.49	−0.02
M-Pu Punitiveness	2.42 (1.30)	2.53 (1.11)	−0.5	0.06
F-Pu Punitiveness	2.70 (1.29)	2.23 (1.02)	2.19^*^	−0.09
M-As Approval-seeking	2.64 (1.32)	3.52 (1.11)	−3.99^***^	0.20^†^
F-As Approval-seeking	2.58 (1.33)	3.47 (1.09)	−3.96^***^	0.11

*** *p≤0.001*;

** *p≤0.01*;

* *p≤0.05*;

† *p≤0.10*.

*n.a., not available. YSQ-S subscales in patient group are based on n=84 with exception of Ed, Ab, Ma, and Si (*n*=83). YPI subscales are based on n=73–83. YSQ-S scores for norm group are retrieved from Stopa and Waters (2005) for YPI from Atalay et al. (2008)*.

As expected, subscales of YSQ-S correlated positively with conceptually corresponding subscales of YPI. Thus, patients’ self-schemas in relation to people in general (e.g., “I worry that people I feel close to will leave me or abandon me”) tended to be congruent with their current self-schema of caregiving experiences during their childhood (e.g., “Withdrew or left me alone for extended periods”). This was true for both experiences with father and with mother (Table 3). Also as expected, negative memories of relational experience of close caregivers assessed by YPI were associated with insecure attachment, particularly with an anxious and fearful-avoidant attachment style. Overall, current self-schema of caregiving experiences during childhood related most strongly to attachment style to mother, and negative experiences with mother were more strongly associated with patients’ attachment style than were their negative experiences with father. Finally, as can be seen in Table 4, negative self-schemas assessed by YSQ-S correlated positively with attachment anxiety and fearful-avoidant attachment.

**Table 3:** Correlations (Pearson *r*) between YSQ-S subscales and YPI(M=mother/F=father) subscales.

YSQ-S	Correlation with YPI-M and YPI-F	
	YPI-M	YPI-F
Ed Emotional deprivation	0.53^***^	0.38^***^
Ab Abandonment	0.25^*^	0.10^†^
Ma Mistrust/abuse	0.45^***^	0.38^***^
Ds Defectiveness /shame	0.40^***^	0.29^**^
Fa Failure to achieve	0.35^***^	0.42^***^
Vh Vulnerability to harm & illness	0.27^*^	0.28^*^
Em Enmeshment	0.25^*^	0.19^†^
Sb Subjugation	0.39^***^	0.52^***^

*** *p≤0.001*;

** *p≤0.01*;

* *p≤0.05*;

† *p≤0.10*.

**Table 4:** Correlations (Pearson *r*) between YSQ-S subscales and ECR-RS.

YSQ-S	ECR-RS											
	Avoidance				Anxiety				Fearful-avoidant			
	AvG	AvM	AvF	AvP	AnG	AnM	AnF	AnP	FAG	FAM	FAF	FAP
Ed	0.18	0.36^***^	0.16	0.48^***^	0.40^***^	0.35^***^	0.25^*^	0.46^***^	0.42^***^	0.44^***^	0.25^*^	0.57^***^
Ab	−0.16	0.01	0.05	0.2	0.62^***^	0.46^***^	0.27^*^	0.47^***^	0.41^***^	0.30^**^	0.19	0.42^***^
Ma	0.09	0.08	0.09	0.35^***^	0.52^***^	0.34^**^	0.18	0.41^***^	0.47^***^	0.26^*^	0.16	0.46^***^
Si	0.24^*^	0.24^*^	0.08	0.53^***^	0.52^***^	0.34^**^	0.16	0.47^***^	0.55^***^	0.36^***^	0.14	0.60^***^
Ds	0.31^**^	0.42^***^	0.23^*^	0.51^***^	0.52^***^	0.48^***^	0.19	0.39^***^	0.59^***^	0.56^***^	0.25^*^	0.53^***^
Fa	0.2	0.28^**^	−0.01	0.40^***^	0.45^***^	0.31^**^	0.07	0.28^**^	0.47^***^	0.37^***^	0.03	0.40^***^
Di	0.11	0.09	0.21	0.34^**^	0.37^***^	0.33^**^	0.11	0.25^*^	0.36^***^	0.27^*^	0.2	0.35^***^
Vh	−0.08	0.08	0.1	0.27^**^	0.50^***^	0.39^***^	0.12	0.33^**^	0.36^***^	0.30^**^	0.13	0.37^***^
Em	−0.04	−0.09	0.09	0.26^*^	0.39^***^	0.22^*^	0.21	0.41^***^	0.30^**^	0.09	0.18	0.41^***^
Sb	0.14	0.23^*^	0.17	0.44^***^	0.52^***^	0.40^***^	0.13	0.43^***^	0.50^***^	0.39^***^	0.18	0.52^***^
Ss	−0.02	−0.05	0.08	0.25^*^	0.31^**^	0.28^**^	0.19	0.29^**^	0.24^*^	0.15	0.16	0.33^**^
Ei	0.31^**^	0.2	0.15	0.38^***^	0.27^**^	−0.05	0.05	0.22^*^	0.40^***^	0.09	0.12	0.35^***^
Us	−0.07	0.2	0.15	0.06	0.26^*^	0.36^***^	0.29^**^	0.22^*^	0.18	0.35^***^	0.26^*^	0.18
Et	0.06	0.03	0.16	0.09	0.30^**^	0.29^**^	0.21	0.32^**^	0.28^**^	0.2	0.22	0.26^*^
Is	0.15	0.06	0.28^**^	0.36^***^	0.46^***^	0.26^*^	0.25^*^	0.30^**^	0.46^***^	0.21	0.31^**^	0.39^***^

*** *p≤0.001*;

** *p≤0.01*;

* *p≤0.05*.

*Abbreviations according to Table 1 (ECR-RS) and 2 (YSQ)*.

Among the 84 patients, 30 were categorized as completely abstinent during 12months (mean value 0), and three patients were reported to have repeated relapses with positive urine analyses in each four-month period (mean value 2). The remaining 51 patients had periods of abstinence and periods of relapses during the past 12months (mean value >0 and<2). The mean value for all patients was 0.62. There were no significant correlations between relapse in drug use and age, gender, marital status, or ethnic background. Outcome in treatment was significantly better for patients who were either working or studying (*p*<0.001). No association was seen between relapse and attachment style (Table 1). For YSQ-S, high scores on three subscales (Enmeshment, Emotional inhibition, and Entitlement) were significantly associated with relapses in drug use, high scores on four subscales being marginally associated with relapse (Table 2). For YPI, relapse was significantly associated with Vulnerability to harm and illness (mother and father) and to Insufficient self-control/-discipline (father), and marginally associated with Negativity/pessimism (father), and Approval-seeking (mother; Table 2).

For the demographic variables of age and cultural background, no significant differences were found on attachment style and self-schema. For gender, a significant difference was found on Self-sacrifice, women scoring higher than men (*p*<0.05). There were no differences between women and men in attachment style. In patients on sick-leave/social welfare, the self-schema scores on Social isolation, Emotional deprivation, Self-sacrifice, and Insufficient self-control/-discipline were more negative compared to patients in work or study. The attachment style in patients with no work (employment or study) was characterized by an insecure (high levels of avoidance or anxiety; *p*<0.005) and also fearful-avoidant (high level of both avoidance and anxiety) attachment style related to a partner (*p*<0.001), compared to patient in work/studying.

The self-schema Social isolation was evident in patients without a partner compared to those with a partner (*p*<0.01). The attachment style in patients without a partner was characterized as high levels of avoidant (*p*<0.001) and fearful-avoidant (*p*<0.01) to earlier partner, compared to patients in a relationship.

## Discussion

The current study provides a comprehensive account of self-schema and attachment style in a sample of opiate dependent patients in ongoing treatment (OMT). This includes current views of experiences with caregivers during childhood, expectations in close relationships (attachment styles) and maladaptive self-schemas that may evolve as a result of unfavorable early experiences in life. As expected, we found an overrepresentation of insecure attachment and maladaptive self-schemas in the patient group compared to non-clinical groups. Inter-correlations conformed to theoretical predictions and revealed consistent individual differences within the patient group in terms of the negativity of the variables. Only a few self-schemas and no attachment variables correlated significantly with patients’ relapse in drug use during treatment, suggesting that other factors may be more important in determining patients’ response in OMT. Consistent with earlier research examining how attachment security is related to the use of addictive substances (see Fairbairn et al., 2018), the present sample of patients with opioid dependence were found to have a more insecure attachment style than non-clinical controls. The mean ECR-RS values in the patient group are significantly higher on all independent scales of attachment avoidance, as well as attachment anxiety, and tend to have values above 3.0, indicating an insecure attachment style (Moreira et al., 2015). Thus, attachment insecurity was documented for close relationships in general, as well as for each one of the assessed specific relationships (mother, father, and romantic partner). It should be noted that, despite this uniform outcome pattern at the group level, variability in attachment style across different significant relationships may still exist at the individual level.

Relational concerns in the patient group are indicated also by their elevated scores (compared to controls) in self-schemas for Mistrust/Abuse, Social isolation, Abandonment, and Enmeshment. Thus, according to our results, opioid dependent patients in OMT may feel a fear of being negatively treated by others, and a lack of positive connectedness and control in social situations. This finding corroborates the results of an earlier study (Brotchie et al., 2004) that revealed significantly higher scores in opiate users compared to non-clinical controls in six self-schemas involving a strong relational component, including three of those found to be significant in the current study: Mistrust/Abuse, Social isolation, and Enmeshment. In several separate self-schemas (Vulnerability to harm and illness, Subjugation, and Emotional inhibition), patients in the present study had values comparable to those of the comparison group. It should be noted that Brotchie et al. (2004) demonstrated that these maladaptive self-schemas occur to a larger extent in patients with alcohol use disorders, compared to opioid use disorders. It has been assumed that patients with opioid use disorders may have a lower grade of awareness related to their self-schemas, and that opioid misuse may contribute to the reinforcement of schema-avoidance (Brotchie et al., 2004). Stopa and Waters (2005) demonstrated that certain self-schemas (Emotional deprivation, Defectiveness/Shame, and Entitlement) may be affected by mood. In the present study, no differences could be seen between clinical patients and a normative group with respect to these self-schemas.

Also, the high mean values for avoidant attachment style are in accordance with previous studies indicating an insecure-avoidant attachment style in patients with illicit drug use disorders (Schindler, 2019). Also, the higher values seen in current self-schemas for Mistrust/abuse, Social isolation, Abandonment, and Enmeshment, compared to a normative group, can describe patients’ experience of isolation. Childhood memories of abandonment and mistrust/abuse (both in relation to mother and father) are consistent with those adulthood consequences.

The present study demonstrated that patients in current work (employment or studying) displayed a higher level of functioning than patients without work. It may not be surprising that maladaptive self-schemas such as social isolation, self-sacrifice, emotional deprivation and insufficient self-control/−discipline are common self-schemas in individuals without a current employment. The attachment style in patients without an employment is characterized by an insecure (including high levels of avoidance or anxiety) and fearful-avoidant attachment style related to one’s partner, and this can be assumed to be related to maladaptive self-schemas. One interesting finding is the fact that attachment style in individuals whose marital status was described as single is characterized by an avoidant and fearful-avoidant attachment, which may indicate that patients who are single tend to have destructive experience of previous relationships.

In those self-schemas that do not explicitly focus on self in relation to others, patients in the current study had values comparable to those of the normative group. Thus, for example, the two groups did not differ significantly in exaggerated or rigid internalized standards of behavior and performance, or in beliefs that one has failed in important areas of achievement. Similarly, Brotchie et al. (2004) found significant differences between opiate users and controls in only one self-schema (insufficient self-control) that lacks an explicit focus on self in relation to others.

Overall, patients reported more negative experiences during upbringing than did controls. This was true both for experiences in relation to mother and experiences in relation to fathers. Consistent with claims that insecure attachment styles have a root in early interactions with caregivers, within the patient group, negative memories of relational experience of close caregivers related strongly to an anxious and fearful-avoidant attachment style. Furthermore, consistent with claims that maladaptive self-schemas are rooted in negative childhood experiences, the present study demonstrated strong associations between self-schemas and memories of relational experience both with mother and father during childhood. Thus, patients’ self-schemas in relation to people in general (e.g., I worry that people I feel close to will leave me or abandon me) tended to be congruent with their current self-schema of caregiving experiences during their childhood (e.g., Withdrew or left me alone for extended periods). Finally, self-schema subscales correlated significantly with attachment scales, and, in particular, with an anxious or fear-avoidant attachment style (Table 4). These associations not only likely reflect a common ground in negative childhood experiences but may also to some extent reflect a conceptual overlap between variables.

Although the results of the current study suggest that relational concerns may contribute to opiate abuse, there was a fairly weak link between such concerns and relapse in drug use during treatment. First, neither attachment avoidance, nor attachment anxiety, correlated significantly with this variable. Second, only a few current views of experiences with caregivers during childhood (vulnerability to harm and illness; insufficient self-control/-discipline) were significantly related to this treatment outcome variable. Finally, among self-schemas, relapse in drug use correlated positively with Entitlement (beliefs that one is superior to others), Emotional inhibition, and Enmeshment (insufficient individual identity), suggesting that core beliefs about the self and self-control may influence treatment outcome. However, these associations need to be corroborated in future research. In addition, as the present study demonstrated that patients in current work (employment or studying) were less likely to relapse into drug abuse than patients who did not work or study, it may be fruitful to investigate whether this variable mediates or moderates the influence of self-schemas and attachment styles on treatment outcomes. It may not be surprising that maladaptive self-schemas such as social isolation, self-sacrifice, emotional deprivation, and insufficient self-control/-discipline are the most pronounced self-schemas in individuals without a current employment. The attachment style in patients without an employment is characterized by an insecure and even fearful-avoidant attachment style related to one’s partner, and this can be assumed to be related to maladaptive self-schemas. One interesting finding is the fact that attachment style in individuals whose marital status was described as single is characterized by an avoidant and fearful-avoidant attachment, which may indicate that patients who are single tend to have destructive experience of previous relationships.

## Limitations and Suggestions For Future Research

The present study relies heavily on self-reports. The validity of these reports may have been lowered by personal factors that bias the recall and communication of personally relevant information such as anxiety and avoidance. In addition, the reporting of memories from the childhood may be affected by more recent experience of the parents’ behavior. Moreover, the fact that some patients remained in active substance use due to medical treatment may have affected their responses to the questionnaire.

Another limitation is the fact that available reference samples for the instruments included are non-clinical samples. While our patient group had a majority of male participants, females were in majority in all three comparison groups. On the other hand, there was only one minor difference (the item self-sacrifice) between women and men in our study. Moreover, two of the comparison groups were quite small. Finally, the normative group described by Fraley et al. (2015), with respect to ECR-RS data, was recruited through an online service, such that is unclear whether this sample entirely constitutes a non-clinical population. For future studies, more research in non-clinical samples is needed, in order to obtain adequate normative data. Also, the studies of attachment styles and self-schemas in different clinical populations, defined by different disorders, are needed in future research.

Because the patients that took part in the study basically received pharmacological treatment, relapse and abstinence in drug abuse during a 1-year period may be regarded as a relatively satisfactory measure of treatment outcome. Future studies might add psychosocial outcome factors such as effects on work, studies, and relationships. Currently, psychological treatment administered by professional psychologists often constitutes only a small part of the activities in opiate maintenance treatment units. More knowledge about this group of patients is needed in order to better outline the difficulties and opportunities for psychological treatment. For example, increased knowledge about attachment styles, self-schemas, and other factors related to vulnerability may be used in the allocation of patients to different family-based treatment approaches and psychotherapeutic interventions (cf. Shorey et al., 2013, 2015; Ghandehari and Dehghani, 2018).

## Data Availability Statement

Data can be made available in case of a formal request from the authors to the ethics committee. Requests to access the datasets should be directed to anders_c.hakansson@med.lu.se.

## Ethics Statement

The studies involving human participants were reviewed and approved by the Regionala Etikprövningsnämnden/Regional Ethics Review Board, Lund, Sweden. The patients/participants provided their written informed consent to participate in this study.

## Author Contributions

EH and EL are responsible for the original conception, data analysis, and writing of the manuscript. EH collected the data. HB and AH supervised the study and made additional comments. All authors contributed to the article and approved the submitted version.

## Conflict of Interest

AH has a research position at Lund University, who is sponsored by an overall study grant from the state-owned Swedish gambling operator AB Svenska Spel and receives study support from the state-owned alcohol monopoly Systembolaget’s research council and from the regional hospital organization in southern Sweden. None of these organizations had any influence on or role in the present research.

The remaining authors declare that the research was conducted in the absence of any commercial or financial relationships that could be construed as a potential conflict of interest.

## Publisher’s Note

All claims expressed in this article are solely those of the authors and do not necessarily represent those of their affiliated organizations, or those of the publisher, the editors and the reviewers. Any product that may be evaluated in this article, or claim that may be made by its manufacturer, is not guaranteed or endorsed by the publisher.
